# Supplementary material for: The causal relationship between the human gut microbiota and pyogenic arthritis: a Mendelian randomization study
Source: Front Cell Infect Microbiol. 2024 Nov 26;14:1452480. doi: 10.3389/fcimb.2024.1452480 (PMC11629706; doi:10.3389/fcimb.2024.1452480)
Supplement: Supplementary file 1 [file DataSheet1.zip › mendelian test/ebi-a-GCST90027711.csv_leave_one_out.pdf]

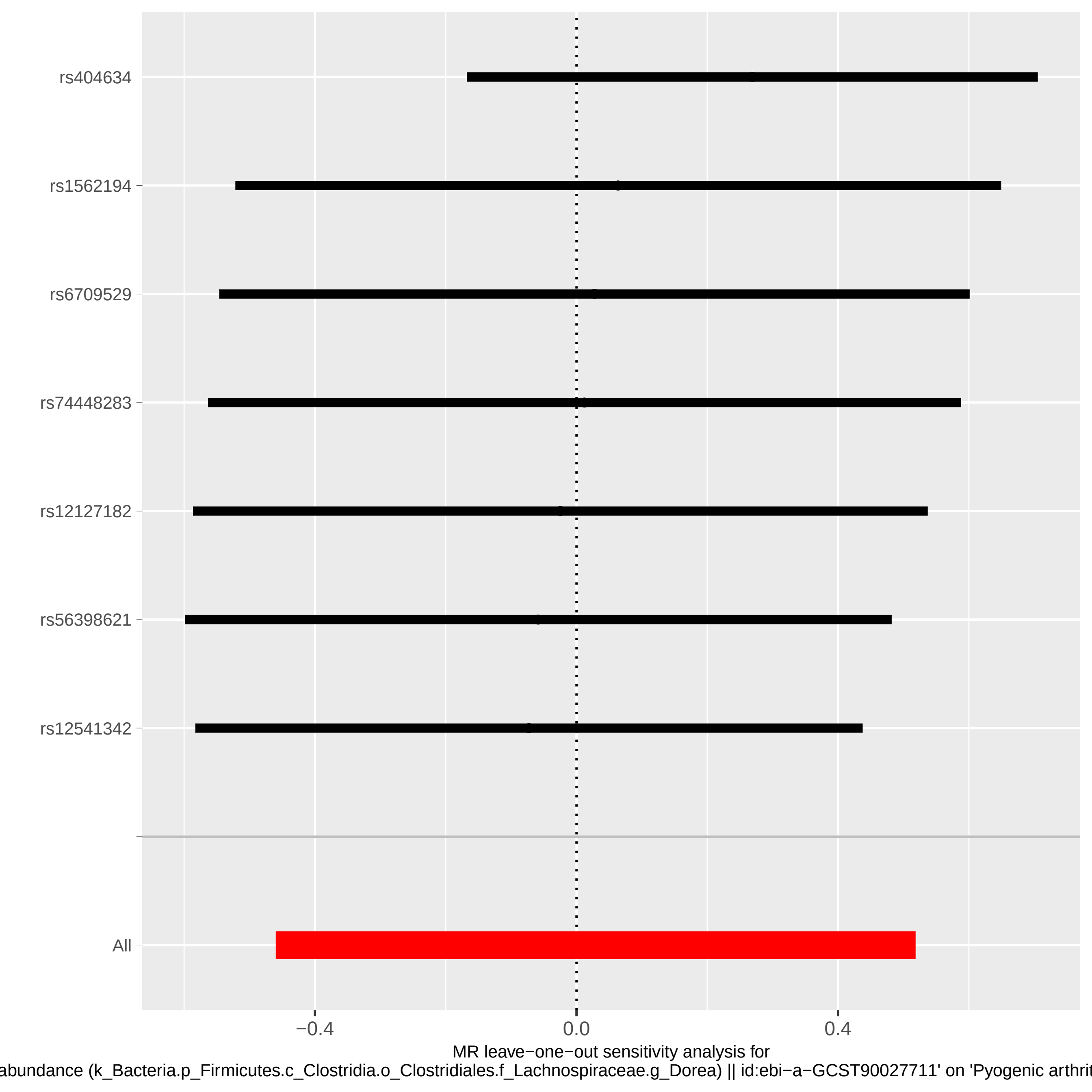
rs404634
rs1562194
rs6709529
rs74448283
rs12127182
rs56398621
rs12541342
All
−0.4
0.0
0.4
MR leave−one−out sensitivity analysis for
abundance (k_Bacteria.p_Firmicutes.c_Clostridia.o_Clostridiales.f_Lachnospiraceae.g_Dorea) || id:ebi−a−GCST90027711' on 'Pyogenic arthrit
